# Ternary Organic Photovoltaics at a Turning Point: Mechanistic Perspectives on Their Constraints

**DOI:** 10.3390/nano15221702

**Published:** 2025-11-11

**Authors:** Hou-Chin Cha, Kang-Wei Chang, Chia-Feng Li, Sheng-Long Jeng, Yi-Han Wang, Hui-Chun Wu, Yu-Ching Huang

**Affiliations:** 1 College of Engineering, Ming Chi University of Technology, New Taipei City 243303, Taiwan; 2 Organic Electronics Research Center, Ming Chi University of Technology, New Taipei City 243303, Taiwan; 3 Department of Materials Engineering, Ming Chi University of Technology, New Taipei City 243303, Taiwan; 4 Department of Materials Science and Engineering, National Taiwan University, Taipei 10617, Taiwan; 5 Department of Material Research, National Atomic Research Institute, Taoyuan 325207, Taiwan; 6 Center for Sustainability and Energy Technologies, Chang Gung University, Taoyuan 33302, Taiwan

**Keywords:** ternary organic photovoltaics, indoor photovoltaic, spectral overlap, carrier mobility, recombination

## Abstract

Ternary organic photovoltaics (OPVs) are considered as the next step beyond binary systems, aiming to achieve synergistic improvements in absorption, energetic alignment, and charge transport. However, despite their conceptual appeal, most ternary blends do not outperform binary counterparts, particularly under indoor illumination where photon flux and carrier dynamics impose strict limitations. To comprehensively understand this discrepancy, multiple ternary systems were systematically examined to ensure that the observed behaviors are representative rather than case specific. In this study, we systematically investigate this discrepancy by comparing representative donor–donor–acceptor (D–D–A) and donor–acceptor–acceptor (D–A–A) systems under both AM 1.5G and TL84 lighting. In all cases, the broadened absorption fails to yield effective photocurrent; instead, redundant excitations, reduced driving forces for charge separation, and disrupted percolation networks collectively diminish device performance. Recombination and transient analyses reveal that the third component often introduces energetic disorder and trap-assisted recombination instead of facilitating beneficial cascade pathways. Although the film morphology remains smooth, interfacial instability under low-light conditions further intensifies performance losses. The inclusion of several systems allows the identification of consistent mechanistic trends across different ternary architectures, reinforcing the generality of the conclusions. This work establishes a mechanistic framework linking molecular miscibility, energetic alignment, and percolation continuity to device-level behavior, clarifying why ternary strategies rarely deliver consistent efficiency improvements. Ultimately, indoor OPV performance is determined not by spectral breadth but by maintaining balanced charge transport and stable energetic landscapes, which represents an essential paradigm for advancing ternary OPVs from concept to practical application.

## 1. Introduction

Organic photovoltaic (OPV) research has long relied on binary active layers as the baseline under both 1-sun and indoor illumination. The advent and continuous optimization of non-fullerene acceptors (NFAs) [1,2,3,4,5,6,7,8,9,10,11] along with improved processing compatibility, have enabled the emergence of ternary architectures designed to overcome the intrinsic limitations of binary systems through three synergistic mechanisms: spectral complementarity to broaden the absorption window, cascade or alloyed energetics to reduce interfacial losses and stabilize the open-circuit voltage (V_OC_); and morphology and transport control helps maintain balance carrier mobilities and suppress recombination. Numerous studies have demonstrated clear gains from binary-to-ternary transitions, confirming the practical value of this approach. Under indoor lighting, devices experience lower photon flux, a narrower spectrum concentrated in the visible range, and sub-1-sun operating intensities, which make carrier generation, dissociation, and extraction more fragile than under sunlight. In this regime, spectral narrowing and non-radiative losses more strongly depress V_OC_, while transport imbalance and limited percolation cap the short-circuit current (J_SC_) and fill factor (FF). Given these constraints, ternary design is well-suited for indoor OPVs because spectral complementarity broadens the usable spectrum, cascade energetics mitigates voltage losses, and morphology and transport control maintain balanced, continuous pathways for charge collection [4,12,13].

Since typical donor (D)–acceptor (A) pairs do not fully cover the visible spectrum, ternary blends with D1:D2:A or D:A1:A2 compositions are adopted to extend absorption and harvest more photocurrent. Selecting the third component is therefore critical. First, match absorption to indoor spectra (≈300–800 nm). Second, raise external quantum efficiency and suppress recombination at low carrier density to avoid J_SC_ losses. Third, favor energetic cascades with the host, often using a wide-bandgap additive, to minimize energy loss and boost V_OC_. Representative cases include adding PDTSTPD to PCDTBT:PC_71_BM, which increases the power conversion efficiency (PCE) from 16.5% to 20.8% via defect suppression and improved transport [14]. A separate study compares D1:D2:A with D:A1:A2 using PBDB-T:PTB7-Th:PC_71_BM and PBDB-T:ITIC-Th:PC_71_BM, respectively [15]. Under 1-sun, the PCEs are similar (9.11% and 9.34%). Under 1000 lux light-emitting diode (LED), however, the D:A1:A2 device reaches 22.73% compared with 18.99% for D1:D2:A, with a clearer cascade lowering the required driving force for charge transfer and reducing trap density. Likewise, inserting Y-Th2 between PM6 and Y6 forms a well-defined cascade, mitigates V_OC_ loss, and yields a uniform fibrous texture with controlled phase separation, achieving 22.7% PCE at 1000 lux LED [16]. These results emphasize suppressing trap-assisted recombination and energetic losses for indoor operation, where carrier loss is more severe than under sunlight [4,9]. Morphology engineering with non-fullerene acceptors can further reduce recombination by improving orientation and phase separation [17]. For example, a D:A1:A2 system (PM6 with ITIC-4F and ITIC-Th) leverages strong acceptor–acceptor compatibility to increase order, with higher shunt resistance, lower leakage, improved V_OC_ and FF, and a final performance of FF = 75.58% and PCE = 28.02% at 1000 lux LED [18]. Collectively, indoor spectral matching, cascade alignment, and controllable morphology and compatibility must all be satisfied to enable consistent improvements in V_OC_, J_SC_, and FF.

However, broader absorption alone does not guarantee usable photocurrent under indoor conditions. When the third component is poorly chosen, it can introduce subtle energetic mismatches that reduce the driving force for charge separation, miscibility boundaries that disrupt molecular packing, or interrupted percolation that breaks continuous transport pathways. These issues manifest as charge-transfer (CT) states and redundant excitations that are harvested inefficiently, leading to concurrent degradations in V_OC_, J_SC_, and FF. In practice, these failure modes show clear diagnostic signatures: stronger bimolecular recombination at elevated local carrier densities, dominant trap-assisted recombination, and atomic force microscope (AFM) evidence of unchanged macroscopic roughness, all indicating molecular-scale origins rather than gross morphology. Narrow miscibility windows can also compromise stability and reproducibility, making performance sensitive to small processing variations. Therefore, absorption and energetic pre-screening is necessary but insufficient; ultimate performance hinges on molecular packing, miscibility, interfacial states, and percolation continuity, which must be clarified through mechanism-oriented characterizations [4,9,12,13,17].

Accordingly, this work advances a mechanism-driven and reproducible framework for ternary OPV. We directly compare D–D–A and D–A–A architecture under a standard solar spectrum (air mass 1.5 global, AM 1.5G) and an indoor illumination (TL84) with controlled materials and processing, and we determine how energetic alignment, molecular compatibility, and percolation continuity govern device-level outcomes (V_OC_, J_SC_, FF, and external quantum efficiency (EQE)). Rather than chasing nominal peak PCE, we identify the operating space in which a third component avoids recombination penalties and transport imbalance and translates spectral gains into usable performance. To substantiate causality, we couple device metrics with targeted diagnostics that probe recombination pathways, carrier extraction and decay, charge-carrier mobility balance, and morphology, while keeping the analysis focused on the governing mechanisms. The result is a verifiable causal map that links material selection and blend topology to performance limits under both 1-sun and indoor conditions, explaining why material-level changes in spectra, energetics, and morphology/transport may or may not yield overall performance gains.

## 2. Experimental Sections

### 2.1. Materials

Indium-doped tin oxide (ITO) coated glass was obtained from Luminescence technology (Lumtec, New Taipei City, Taiwan). Zinc acetate dihydrate (≥98%), ethanolamine (MEA, 98%), 2-methoxyethanol (2-ME, 99.8%), o-Xylene (≥99.8%), 2-propanol (99.5%) and acetone (99.5%) were purchased from Sigma Aldrich (St. Louis, MO, USA). The donor polymers and acceptors used in this study were obtained from commercial sources and used as received without further purification. Poly[(2,6-(4,8-bis(5-(2-ethylhexyl-3-fluoro)thiophen-2-yl)-benzo [1,2-b:4,5-b′]dithiophene))-alt-(5,5-(1′,3′-di-2-thienyl-5′,7′-bis(2-ethylhexyl)benzo [1′,2′-c:4′,5′-c′]dithiophene-4,8-dione))] (PM6) was purchased from 1-Material. Poly [4,8-bis(5-(2-ethylhexyl)thiophen-2-yl)benzo [1,2-b;4,5-b′]dithiophene-2,6-diyl-alt-(4-(2-ethylhexyl)-3-fluorothieno [3,4-b]thiophene-)-2-carboxylate-2,6-diyl)] (PTB7-Th), poly[(2,6-(4,8-bis(5-(2-ethylhexyl)thiophen-2-yl)-benzo [1,2-b:4,5-b′]dithiophene))-alt-(5,5-(1′,3′-di-2-thienyl-5′,7′-bis(2-ethylhexyl)benzo [1′,2′-c:4′,5′-c′]dithiophene-4,8-dione))] (PBDB-T), 3,9-bis(2-methylene-((3-(1,1-dicyanomethylene)-6,7-difluoro)indanone))-5,5,11,11-tetrakis(4-hexylphenyl)-dithieno [2,3-d:2′,3′-d′]-s-indaceno [1,2-b:5,6-b′]dithiophene (ITIC-4F), 2,2′-[[12,13-bis(2-ethylhexyl)-12,13-dihydro-3,9-diundecylbisthieno [2″,3″:4′,5′]thieno [2′,3′:4,5]pyrrolo [3,2-e:2′,3′-g][2,1,3]benzothiadiazole-2,10-diyl]bis[methylidyne(5,6-difluoro-3-oxo-1H-indene-2,1(3H)-diylidene)]]bis[propanedinitrile] (Y6), 3TT-FIC, and 4,4,10,10-tetrakis(4-hexylphenyl)-5,11-(2-ethylhexyloxy)-4,10-dihydrodithienyl [1,2-b:4,5-b′]benzodithiophene-2,8-diyl)bis(2-(3-oxo-2,3-dihydroinden-5,6-dichloro-1-ylidene)malononitrile) (BT-CIC) were supplied by commercial vendors. The fullerene derivative (6,6)-phenyl C_71_ butyric acid methyl ester (PC_71_BM, mixture of isomers) was purchased from Lumtec (New Taipei City, Taiwan). Molybdenum (VI) oxide (MoO_3_), (99.9995%) was purchased from Alfa Aesar (Ward Hill, MA, USA). Ag was purchased from Gredmann Taiwan Ltd. (New Taipei City, Taiwan).

### 2.2. Device Fabrication

Indium tin oxide (ITO) coated glass substrates (15 Ω/square) were sequentially cleaned by ultrasonication in detergent, deionized water, acetone, and isopropanol for 30, 10, 10, and 30 min, respectively, then dried under a nitrogen stream and treated with UV–ozone irradiation to remove residual organic contaminants and improve surface wettability. A zinc oxide (ZnO) sol–gel precursor solution was spin-coated at 3000 rpm for 30 s onto the pre-cleaned ITO substrates, followed by thermal annealing at 180 °C for 20 min in air to form the electron transport layer (ETL). Different active layer formulations were then deposited onto the ZnO layer: PM6:PBDB-T:IT-4F was spin-coated at 3000 rpm for 30 s and annealed at 100 °C for 10 min; PCDTBT:PM6:PC_71_BM was spin-coated at 1000 rpm for 30 s; PM6:Y6:PC_71_BM was spin-coated at 5000 rpm for 30 s; PTB7-Th:3TT-FIC:PC_71_BM was spin-coated at 4000 rpm for 30 s; and PM6:BT-CIC:IT-4F was spin-coated at 5000 rpm for 30 s followed by annealing at 100 °C for 10 min. After active layer deposition, substrates were transferred into a high-vacuum thermal evaporation chamber (base pressure ~2 × 10^−6^ Torr), where 5 nm of molybdenum(VI) oxide (MoO_3_) was deposited at 0.1 Å/s as the hole transport layer (HTL), followed by 100 nm of Ag deposited at 1.5 Å/s as the top electrode. The resulting device structure was ITO/ZnO/active layer/MoO_3_/Ag, with an active area of 0.04 cm^2^ defined by a shadow mask during electrode deposition.

### 2.3. Characterization

Current density–voltage (J–V) characteristics of the photovoltaic devices were measured using a source meter (Keithley 2410) (Keithley Instruments Inc., Cleveland, OH, USA) under simulated AM 1.5G illumination (100 mW cm^−2^) provided by a solar simulator SS-X100R (Enli Technology Co., Ltd., New Taipei City, Taiwan), calibrated with a certified silicon reference cell. Indoor performance was evaluated by an integrated system (Industrial Technology Research Institute, ITRI) composed of a power meter (Keithley 2401) (Keithley Instruments Inc., Cleveland, OH, USA) and a light source of Philips TLD 18W/830 (Philips Lighting, Eindhoven, The Netherlands). External quantum efficiency (EQE) spectra were recorded using a quantum efficiency measurement system QE-R3011 (Enli Technology Co., Ltd., New Taipei City, Taiwan), and the integrated J_SC_ values were cross-checked with those obtained from J–V curves. Optical absorption spectra of the active layers were measured using a UV–Vis–NIR spectrophotometer V-650 (JASCO Corporation, Tokyo, Japan). Morphological characterization was carried out by atomic force microscopy (AFM, Bruker Corporation, Billerica, MA, USA) in tapping mode to determine the root-mean-square (RMS) surface roughness. The work function of the films was assessed using a scanning Kelvin probe SKP5050 (KP Technology Ltd., Wick, Scotland, UK), enabling precise evaluation of surface potential and work function variations. Transient photocurrent (TPC), transient photovoltage (TPV), and photo-induced charge extraction by linearly increasing voltage (Photo-CELIV) were performed using an all-in-one system (PAIOS, Fluxim AG, Winterthur, Switzerland). Charge transport parameters including carrier mobility and extraction time were extracted from these measurements. In addition, light-intensity-dependent J–V measurements were conducted using neutral density filters to analyze recombination mechanisms via the dependence of V_OC_ and J_SC_ on incident light power.

## 3. Results and Discussion

This study examines whether specific material choices in ternary systems can be translated into measurable performance gains. D–D–A and D–A–A blends were prepared under identical processing and evaluated under AM 1.5G and TL84 indoor illumination. Changes in spectra, energetics, and morphology/transport were tracked to establish causal links between energetic alignment, molecular compatibility, percolation continuity, and the device metrics including V_OC_, J_SC_, FF, and EQE. We evaluate whether gains from spectral broadening and cascade alignment appear only with balanced, continuous transport; otherwise, CT states, trap-assisted recombination, and disrupted percolation nullify them. The following sections establish the conditions under which a third component delivers overall performance gains rather than merely altering the active layer.

### 3.1. Ternary D–D–A System

**Figure 1:**
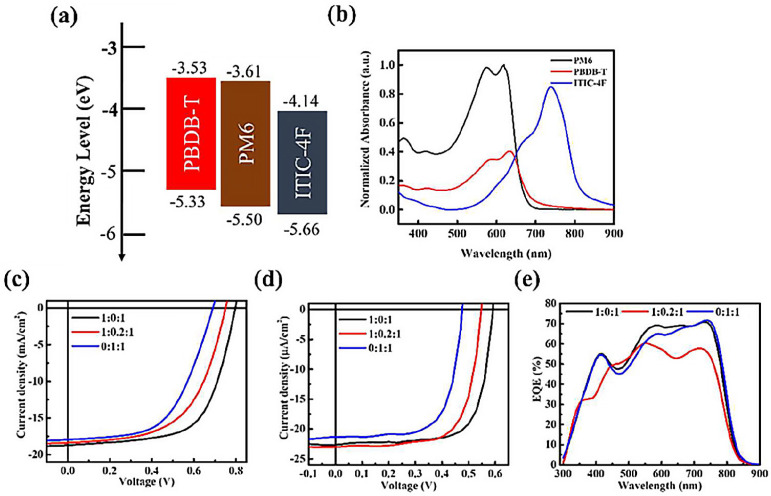
(**a**) Energy level diagram and (**b**) absorption spectra of the active layer materials PBDB-T, PM6, and ITIC-4F. J–V characteristics of devices with different PM6:PBDB-T:ITIC-4F ratios under (**c**) solar illumination and (**d**) indoor lighting, and (**e**) the corresponding EQE spectra.

The ternary PM6:PBDB-T:ITIC-4F system was selected to investigate the donor–donor–acceptor (D–D–A) configuration under both solar and indoor illumination, with PM6:ITIC-4F serving as the binary system due to its well-established performance, and PBDB-T incorporated as a secondary donor. The energy levels of PBDB-T are reasonably aligned with those of PM6 and ITIC-4F (Figure 1a), suggesting the possibility of cascade-assisted charge transfer. In addition, PBDB-T provides an absorption shoulder in the 550–650 nm region (Figure 1b), which could extend the spectral response of the device. However, since this region overlaps strongly with that of PM6, the intention of introducing PBDB-T was not to increase photocurrent directly but to test whether donor–donor mixing could mitigate recombination losses and stabilize performance, particularly under low-intensity indoor conditions. The current–voltage (J–V) results under both standard solar illumination and indoor light are shown in Figure 1c,d and summarized in Table 1. It is evident that the addition of PBDB-T does not enhance the photocurrent, consistent with expectations. On the contrary, with 20% PBDB-T, V_OC_ decreases under both light sources, from 0.79 V to 0.75 V under AM 1.5G illumination, and from 0.60 V to 0.55 V under TL84 indoor light. The corresponding EQE spectra (Figure 1e) reveal that the extra absorption from PBDB-T is not effectively converted into photocurrent; instead, a consistent reduction in EQE is observed across the visible region. These findings highlight a fundamental limitation of D–D–A blending. Although the optical absorption appears broadened, there is no genuine spectral complementarity between PBDB-T and PM6. Excitons generated in PBDB-T are not efficiently dissociated at the donor–acceptor interface. Instead, PM6 and PBDB-T compete for exciton transfer to ITIC-4F, resulting in exciton crowding, extended exciton lifetimes within donor domains, and a higher likelihood of bound-pair recombination and trap-assisted recombination. Moreover, because PM6 and PBDB-T possess similar HOMO levels, the driving force for charge separation is reduced, destabilizing energy-level alignment. This creates suboptimal interfacial states that promote non-radiative recombination pathways. The photovoltaic parameters in Table 1 corroborate these mechanisms, showing concurrent losses in V_OC_, J_SC_, and FF. Under indoor illumination, where photon flux is inherently low and the spectrum is narrowly distributed, these deficiencies become even more pronounced. Redundant absorption provides no net photocurrent gain, while even minor recombination channels substantially reduce both V_OC_ and J_SC_, reflecting the fragile balance of carrier generation, dissociation, and extraction at low light intensity.

**Figure 2:**
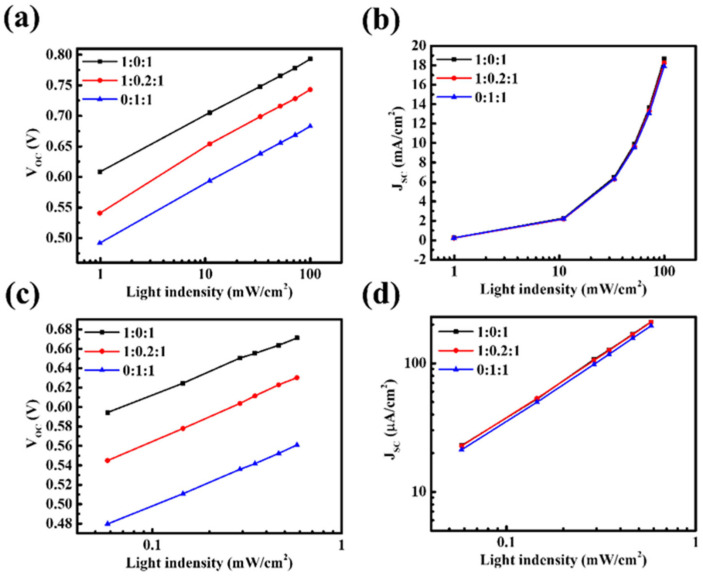
Light-intensity dependence of (**a**) V_OC_ and (**b**) J_SC_ under solar illumination, and (**c**) V_OC_ and (**d**) J_SC_ under indoor illumination for devices with different PM6:PBDB-T:ITIC-4F ratios.

We hypothesize that incorporating PBDB-T into PM6:ITIC-4F increases the likelihood of charge recombination during transport, thereby causing reductions in both V_OC_ and J_SC_. To verify this, light-intensity-dependent measurements were performed to analyze charge recombination behavior. Under both solar and indoor illumination, V_OC_ and J_SC_ were measured as a function of light intensity (P) to differentiate between recombination mechanisms. The relationship between V_OC_ and light intensity reflects trap-assisted (monomolecular) recombination. When the ideality factor n approaches 1, the probability of non-radiative recombination through trap states is low; in contrast, n values significantly greater than 1 indicate pronounced trap-assisted recombination, where charges are more likely to recombine at trap states in the bulk or at interfaces. On the other hand, the dependence of J_SC_ on light intensity is associated with bimolecular recombination. An exponent α close to 1 indicates that photogenerated carriers can be efficiently transported to the electrodes without recombination, whereas α values significantly below 1 suggest that increased carrier accumulation in the active layer enhances bimolecular recombination. The results show that, under both AM 1.5G solar illumination and TL84 indoor light, the n and α values of the ternary system deviate markedly from unity (Figure 2 and Table S1). This indicates the simultaneous presence of severe trap-assisted and bimolecular recombination. The deterioration can be attributed to several mechanistic factors: (i) the energy levels of PBDB-T and PM6 are too close, leading to insufficient driving force for charge separation, unfavorable interfacial states, and enhanced trap-related recombination. (ii) Exciton transfer competition between the two donors extends exciton lifetimes and causes charge accumulation, thereby increasing the probability of bimolecular recombination. (iii) The incorporation of PBDB-T may disrupt the energy level alignment between the active layer and transport layers, further limiting efficient charge extraction. In summary, these results demonstrate that in the ternary D–D–A system, charges cannot be effectively extracted from the active layer but instead suffer from severe recombination losses in both the bulk and interfacial regions, leading to the simultaneous reduction in V_OC_ and J_SC_.

**Figure 3:**
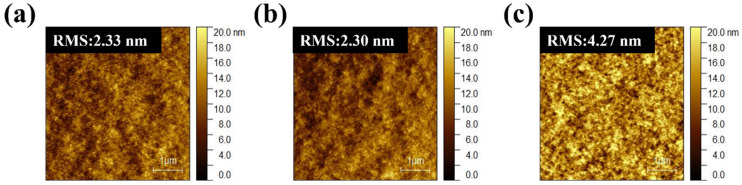
AFM images of PM6:PBDB-T:IT-4F active layers with different ratios: (**a**) 1:0:1, (**b**) 1:0.2:1, and (**c**) 0:1:1.

**Figure 4:**
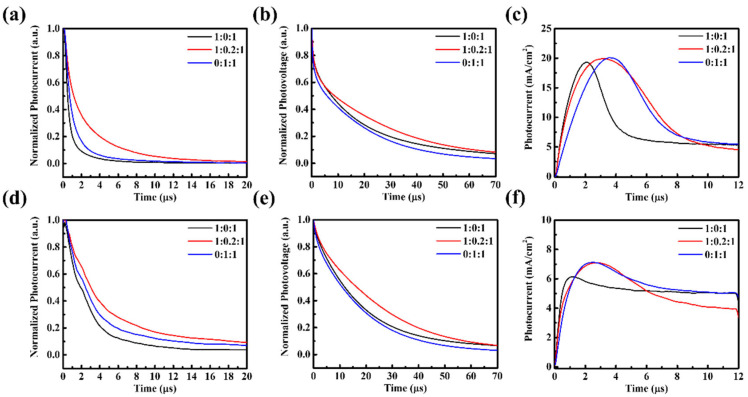
PM6:PBDB-T:IT-4F devices under solar illumination: (**a**) TPC, (**b**) TPV, and (**c**) Photo-CELIV; and under indoor illumination: (**d**) TPC, (**e**) TPV, and (**f**) Photo-CELIV.

AFM analysis (Figure 3) indicates that the surface morphologies of PM6:PBDB-T:IT-4F active layers remain relatively smooth, with RMS roughness values of 2.33, 2.30, and 4.27 nm for different ternary ratios, comparable to the binary control. This excludes large-scale surface roughness or phase segregation as the dominant cause of performance loss. Instead, the deterioration originates from molecular-level interactions. Specifically, partial miscibility between PM6 and PBDB-T may disrupt the optimized packing of PM6, while the introduction of PBDB-T creates unfavorable energetic offsets that promote recombination-prone CT states. TPC, TPV, and Photo-CELIV results (Figure 4) together with the quantitative transport parameters (Table S2) provide direct evidence. Under solar illumination, ternary blends exhibit prolonged charge extraction times (e.g., 1.90 μs for 1:0.2:1 vs. 0.66 μs for the binary) and markedly reduced mobilities (1.25 vs. 3.50 × 10^−5^ cm^2^/Vs), while under indoor light the mobility reduction is nearly an order of magnitude. Such slower extraction indicates that carriers are forced into less favorable percolation pathways, where they are more likely to encounter traps or undergo bimolecular recombination before being collected. Moreover, the imbalance between the two donor networks produces asymmetric transport: PBDB-T contributes to charge generation but fails to form continuous conductive channels, thereby disturbing percolation and amplifying the disparity between electron and hole mobilities. This asymmetry manifests as elongated carrier decay times (up to 24.21 μs for 1:0.2:1 under TL84) and directly translates into reduced FF and suppressed J_SC_. Hence, the efficiency loss in ternary blends is not caused by surface roughness but by disrupted molecular packing, donor–donor miscibility limits, and unfavorable energetic landscapes that collectively hinder balanced charge transport and extraction.

A similar trend was also identified in another D–D–A configuration, namely PCDTBT:PM6:PC_71_BM. Here, the binary PCDTBT:PC_71_BM system absorbs primarily in the 350–650 nm range, while the incorporation of PM6 extends absorption into the 650–800 nm region (Figure S1b). However, the broadened spectral coverage did not lead to improved photocurrent. As shown in the current–voltage characteristics (Figure S1c,d; Table S3), the addition of PM6 initially reduced both V_OC_ and J_SC_ under both solar and indoor illumination. For instance, under 1-Sun, moving from the baseline binary 1:0:2 device (V_OC_ = 0.90 V, J_SC_ = 10.41 mA cm^−2^, FF = 61.84%, PCE = 5.77%) to a ternary blend with 0.7:0.3:2 led to pronounced losses (V_OC_ = 0.88 V, J_SC_ = 9.01 mA cm^−2^, FF = 55.96%, PCE = 4.43%). These losses can be attributed to the increasingly aligned HOMO and LUMO levels of PCDTBT and PM6 at intermediate mixing ratios (Figure S1a), which reduce the energetic offset, diminish the driving force for exciton dissociation, and thereby enhance non-radiative recombination. Interestingly, as the PM6 fraction continued to increase, device performance recovered. At the 0:1:2 endpoint, the restored energetic offset between PM6 and PC_71_BM facilitated more effective charge transfer and reduced recombination, yielding significantly improved characteristics (J_SC_ = 11.52 mA cm^−2^, FF = 70.41%, PCE = 7.32%). A similar trend was observed under TL84 indoor illumination: relative to the binary 1:0:2 device (V_OC_ = 0.67 V, PCE = 14.28%), the ternary 0.7:0.3:2 blend suffered clear deterioration (V_OC_ = 0.57 V, PCE = 9.13%), while the 0:1:2 device again outperformed both baselines, reaching V_OC_ = 0.70 V and PCE = 16.27% (Table S3). Collectively, these results demonstrate that simply broadening the donor manifold does not guarantee higher photocurrent. At intermediate donor–donor mixing ratios, spectral redundancy, reduced energetic offsets, and disturbed transport pathways amplify trap-assisted and bimolecular recombination, leading to suppressed V_OC_, J_SC_, and FF. Only when one donor dominates does the system regain efficient charge separation and balanced transport, explaining why the single-donor endpoints outperform the mixed-donor ternary devices.

By comparing the two representative D–D–A systems, PM6:PBDB-T:ITIC-4F and PCDTBT:PM6:PC_71_BM, a consistent picture emerges highlighting the intrinsic limitations in donor–donor–acceptor ternary configurations. First, spectral overlap between the donors leads to redundant absorption that is not effectively converted into photocurrent. Second, the reduced energetic offset and the presence of multiple exciton transfer pathways decrease exciton dissociation efficiency and increase non-radiative recombination. Third, the charge-transport network becomes disrupted, resulting in reduced carrier mobilities and imbalanced electron–hole transport, which ultimately deteriorate FF and J_SC_. These effects are particularly pronounced under indoor illumination, where the combination of low photon flux and longer carrier lifetimes magnifies the impact of recombination losses. Taken together, these findings demonstrate that the drawbacks observed are not restricted to a specific material set but represent fundamental challenges of the D–D–A architecture itself. Importantly, they also underscore that successful ternary design requires more than spectral complementarity; careful consideration of energy-level alignment, donor–donor miscibility, and percolation continuity is essential if D–D–A blends are to overcome their inherent limitations.

### 3.2. Ternary D–A–A System

**Figure 5:**
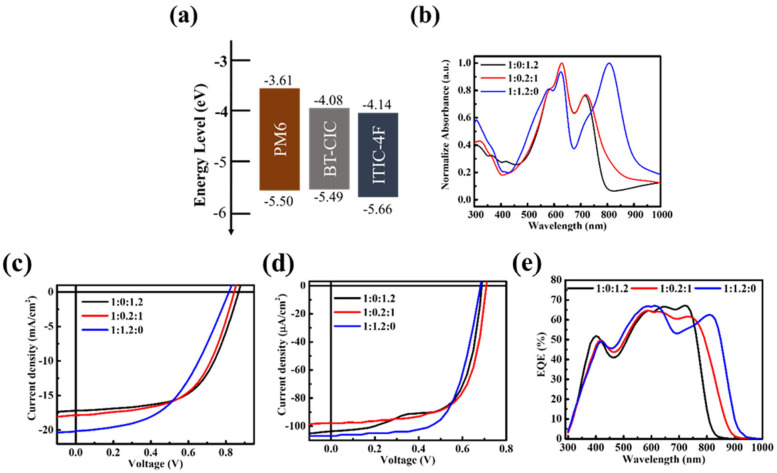
(**a**) Energy-level diagram of the active-layer materials PM6, BT-CIC, and ITIC-4F. (**b**) Absorption spectra of PM6:BT-CIC:ITIC-4F blend films at different composition ratios. (**c**) J–V characteristics under AM 1.5G illumination (100 mW cm^−2^). (**d**) J–V characteristics under TL84 indoor lighting (1000 lux). (**e**) External quantum efficiency (EQE) spectra for the corresponding devices.

The ternary PM6:BT-CIC:ITIC-4F system was designed to examine the donor–acceptor–acceptor (D–A–A) configuration under both solar and indoor illumination, with PM6:ITIC-4F serving as the binary reference, and BT-CIC incorporated as a secondary acceptor. The energy levels of BT-CIC are relatively well aligned with those of PM6 and ITIC-4F (Figure 5a), suggesting a potential cascade pathway for electron transfer from PM6 to BT-CIC and subsequently to ITIC-4F. In addition, BT-CIC exhibits a distinct absorption feature in the near-infrared region (700–800 nm, Figure 5b), which may be expected to extend the spectral response and enhance photocurrent generation. However, as confirmed by the J–V and EQE measurements shown in Figure 5c–e and summarized in Table 2, these anticipated benefits were not observed. The incorporation of BT-CIC does not produce a net photocurrent gain; instead, the ternary devices exhibit performance comparable to or slightly lower than the binary PM6:ITIC-4F reference under AM 1.5G illumination. The EQE spectra reveal that the additional absorption arising from BT-CIC does not contribute effectively to charge generation but instead results in a suppressed spectral response across the visible region. This indicates that excitons generated in BT-CIC are not efficiently dissociated at the donor–acceptor interface due to insufficient energetic offsets. Rather than forming a cascade-assisted charge transfer pathway, BT-CIC introduces redundant excitation channels that promote exciton accumulation and the formation of shallow CT states with limited driving force for separation. These non-ideal CT states act as recombination centers, facilitating non-radiative decay and reducing carrier extraction efficiency. Under TL84 indoor illumination, these effects become even more pronounced, as the reduced photon flux and narrower spectral distribution intensify the imbalance between exciton generation and dissociation. Although V_OC_ remains nearly unchanged, both J_SC_ and FF decrease substantially, leading to a notable drop in overall PCE (from 15.45% to 13.48%).

**Figure 6:**
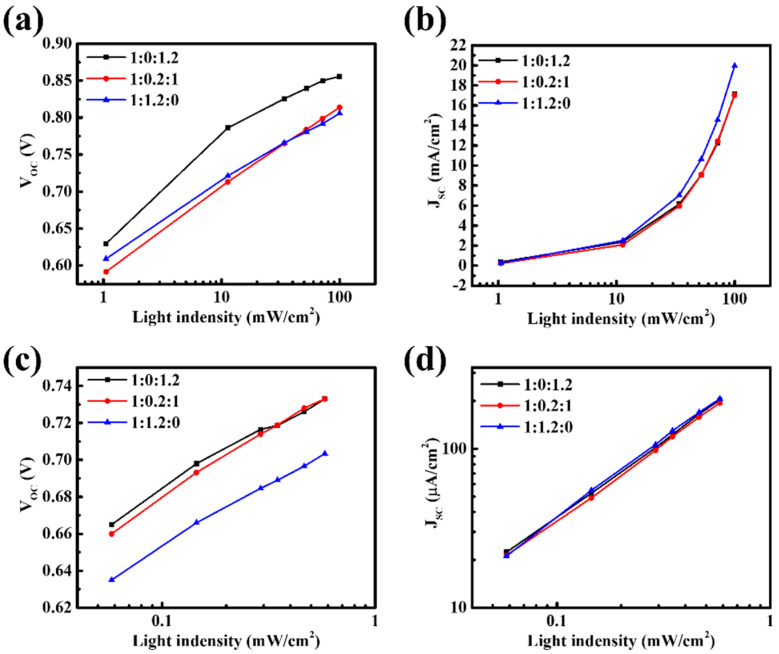
Light-intensity dependence of (**a**) V_OC_ and (**b**) J_SC_ under AM 1.5G, and (**c**) V_OC_ and (**d**) J_SC_ under TL84 indoor illumination for devices with different PM6:BT-CIC:ITIC-4F composition ratios.

Light-intensity-dependent measurements were further conducted to elucidate the recombination behavior of the ternary PM6:BT-CIC:ITIC-4F system (Figure 6, Table S4). Under AM 1.5G illumination, the ternary devices exhibit α values approaching unity, indicating that bimolecular recombination is partially suppressed. This improvement can be ascribed to the formation of additional percolation pathways within the BT-CIC domains, which facilitate carrier transport. However, the consistently high ideality factor n reveals that trap-assisted recombination remains significant. This suggests that while BT-CIC provides auxiliary electron-transport channels, it simultaneously introduces interfacial trap states or perturbs the donor–acceptor energetic alignment, thereby promoting trap-mediated recombination. Under TL84 indoor illumination, the imbalance between these two recombination processes becomes more pronounced. While α remains comparable to that of the binary reference, the n values increase markedly, confirming that carrier trapping dominates over bimolecular recombination under low-light conditions. Such behavior implies the formation of trap-rich interfacial regions or shallow CT states that extend carrier lifetimes but hinder efficient charge extraction. As a result, photogenerated carriers accumulate within localized energy minima and undergo non-radiative decay before reaching the electrodes. Collectively, these results demonstrate that in the D–A–A configuration, the introduction of BT-CIC fails to mitigate recombination losses; instead, it alters the interfacial energetics in a manner that exacerbates trap-assisted processes, thereby accounting for the observed degradation in device performance, particularly under indoor illumination.

**Figure 7:**
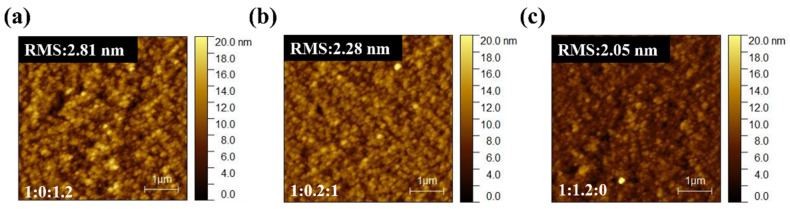
AFM height images of active layers with PM6:BT-CIC:ITIC-4F compositions: (**a**) 1:0:1.2, (**b**) 1:0.2:1, and (**c**) 1:1.2:0.

**Figure 8:**
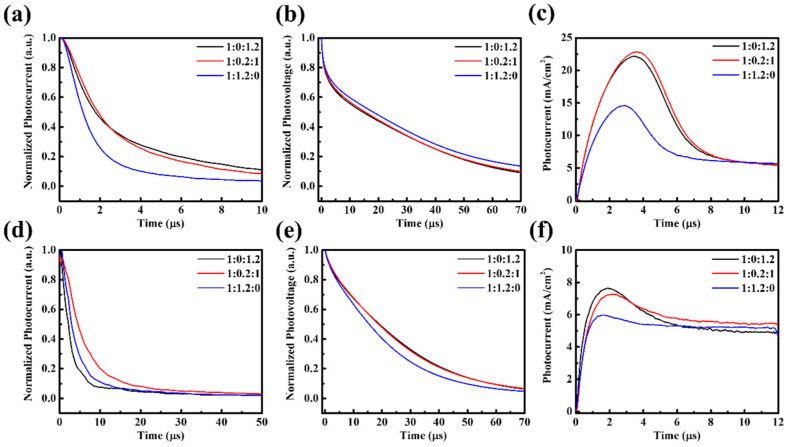
Transient responses for PM6:BT-CIC:ITIC-4F devices: (**a**) TPC, (**b**) TPV, and (**c**) Photo-CELIV under AM 1.5G illumination; (**d**) TPC, (**e**) TPV, and (**f**) Photo-CELIV under TL84 indoor lighting.

AFM analysis (Figure 7) reveals that the surface morphologies of PM6:BT-CIC:ITIC-4F active layers remain smooth and homogeneous, with RMS roughness values ranging from 2.0 to 2.8 nm—comparable to the binary control. This excludes macroscopic phase segregation or surface roughness as the origin of the performance deterioration. Instead, the degradation arises from molecular-scale miscibility and packing disruptions within the acceptor framework. Partial miscibility between BT-CIC and ITIC-4F perturbs the optimized molecular ordering of the host acceptor, producing heterogeneous energetic landscapes and locally disordered percolation pathways that hinder efficient carrier transport. TPC and TPV analyses provide further corroboration (Figure 8, Table S5). Under AM 1.5G illumination, charge extraction and decay dynamics of the ternary devices are similar to those of the binary reference, consistent with their similar efficiencies. Under TL84 indoor light, however, pronounced transport asymmetry emerges. The charge extraction time of the ternary device (5.66 μs) is nearly twice that of the binary (3.01 μs), accompanied by a substantial drop in carrier mobility (2.29 vs. 5.62 × 10^−5^ cm^2^/V s). These results indicate that BT-CIC interrupts continuous electron percolation channels, forcing charge carriers to traverse less favorable transport routes with higher trap densities. As a result, space-charge accumulation intensifies, leading to increased carrier-lifetime asymmetry, reduced fill factor, and enhanced trap-assisted recombination. Therefore, despite its morphological uniformity, the BT-CIC-based ternary blend exhibits intrinsic microstructural and energetic disorder that collectively impede balanced charge transport and extraction, ultimately limiting device performance under low-light operation.

A similar inefficiency was observed in the PM6:Y6:PC_71_BM system. While PM6:Y6 blends already harvest photons up to the near-infrared, PC_71_BM was added to extend visible-light absorption and stabilize V_OC_ (Figure S2). However, PCE consistently decreased upon PC_71_BM incorporation—from 12.52% to 10.73% under AM 1.5G, and from 17.79% to 15.04% under TL84 indoor light (Table S6). The primary degradation pathway was the reduction in FF, likely arising from PC_71_BM aggregation and poor miscibility with the Y6-rich host. Such clustering disrupts percolation, creating isolated domains and charge-blocking regions. As a result, photocurrent generation becomes spatially inhomogeneous, and recombination is amplified. In the PTB7-Th:3TT-FIC:PC_71_BM system, PC_71_BM was added to supplement visible absorption (400–550 nm), complementing the broad but near-infrared-shifted absorption of PTB7-Th:3TT-FIC blends (Figure S3). Under solar illumination, modest improvements were observed when a small fraction of PC_71_BM was incorporated, raising PCE to ~10% at the 1:0.7:0.5 ratio (Figure S4; Table S7). However, when PC_71_BM fully replaced 3TT-FIC, V_OC_ increased (0.78 V) but JSC sharply declined, limiting PCE to only 6.88%. More critically, under TL84 indoor light, ternary devices underperformed compared with binary PTB7-Th:PC_71_BM, with efficiencies peaking at 13.84% but still lower than the expected contribution from spectral broadening. Mechanistically, this discrepancy stems from the mismatch between indoor light spectra and the absorption of 3TT-FIC, which lies predominantly in the near-infrared and thus contributes little indoors. Additionally, excess PC_71_BM induces crystalline rearrangements, lowering FF and introducing transport asymmetry.

Taken together, the results from PM6:BT-CIC:ITIC-4F, PM6:Y6:PC_71_BM, and PTB7-Th:3TT-FIC:PC_71_BM highlight the intrinsic limitations of D–A–A ternary strategies. While the introduction of a second acceptor can nominally broaden optical absorption and, in principle, provide cascade-assisted electron transfer, these anticipated benefits are consistently undermined by redundant excitations, incomplete cascade formation, and destabilized energetic offsets. The competing charge-transfer pathways extend exciton lifetimes and enhance geminate or trap-assisted recombination, while poor miscibility and domain mismatch disrupt percolation networks and introduce transport asymmetry between electrons and holes. These molecular- and mesoscopic-scale instabilities manifest as reduced mobilities, imbalanced carrier extraction, and suppressed V_OC_, J_SC_, and FF. The detrimental effects are particularly severe under indoor illumination, where the combination of low photon flux and long carrier lifetimes magnifies recombination penalties. Collectively, these findings indicate that simply broadening absorption through an additional acceptor is insufficient to achieve performance gains. Instead, successful D–A–A design requires precise control over energy-level alignment, donor–acceptor miscibility, and percolation continuity to prevent recombination-prone CT states and enable efficient charge transport.

By comparatively analyzing five representative ternary systems, two based on donor–donor–acceptor (D–D–A) configurations and three based on donor–acceptor–acceptor (D–A–A) configurations, we can clearly distinguish the mechanistic origins of their limitations. In D–D–A systems, the addition of a second donor introduces spectral redundancy and narrows the driving force for charge separation, while also disturbing percolation pathways and creating transport asymmetry. In D–A–A systems, the inclusion of a secondary acceptor extends absorption into the near-infrared and in principle offers cascade-assisted electron transfer, but in practice it generates non-ideal CT states, energetic disorder, and disrupted electron percolation due to poor miscibility with the primary acceptor. Despite these mechanistic differences, both architectures converge to similar device-level outcomes: enhanced recombination, transport imbalance, and reduced photovoltaic parameters under both solar and indoor illumination. The comparative summary is presented in Table 3.

## 4. Conclusions

This work demonstrates that ternary strategies for indoor OPVs face intrinsic challenges that often overshadow their apparent optical benefits. A comparative investigation of several ternary systems, including both D–D–A and D–A–A types, revealed that the addition of a third component broadened absorption but failed to generate useful photocurrent, instead introducing energetic disorder, redundant excitations, and competing charge-transfer pathways. These effects diluted the driving force for exciton dissociation, created recombination-prone interfacial states, and disrupted charge percolation networks, leading to consistent losses in V_OC_, J_SC_, and FF. The penalties became even more severe under indoor illumination, where low photon flux and long carrier lifetimes magnified the impact of trap-assisted and bimolecular recombination. Taken together, these results highlight an important conclusion: for indoor OPVs, efficiency is not limited by insufficient absorption breadth but by the stability of energetic alignment and the continuity of charge-transport pathways. Unless the third component delivers truly orthogonal absorption, well-defined cascade energetics, and preserves transport balance, ternary blending is more likely to degrade than to enhance performance. The observed behaviors across different ternary systems confirm that these findings are broadly applicable, rather than limited to a specific set of materials. This underscores the need for stricter material selection and interfacial design rules if ternary strategies are to evolve beyond proof-of-concept and deliver reliable gains under realistic indoor conditions.

## Figures and Tables

**Table 1:** Photovoltaic characteristics of devices with different PM6:PBDB-T:ITIC-4F ratios under various illumination conditions. The reported averages (mean ± standard deviation) were obtained from 10 devices.

Light Source	Ratio	V_OC_ (V)	J_SC_ (mA/cm^2^)	FF (%)	PCE (%) (Max)
Sun	1:0:1	0.79 ± 0.00	18.73 ± 0.03	60.88 ± 2.65	9.05 ± 0.39 (9.45)
	1:0.2:1	0.75 ± 0.00	18.39 ± 0.15	53.35 ± 2.49	7.36 ± 0.33 (7.87)
	0:1:1	0.69 ± 0.01	17.66 ± 0.39	54.88 ± 2.09	6.72 ± 0.35 (6.97)
**Light Source**	**Ratio**	**V_OC_** **(V)**	**J_SC_** **(μA/cm^2^)**	**FF** **(%)**	**PCE** **(%) (Max)**
TL84 (200 lux)	1:0:1	0.60 ± 0.00	22.82 ± 0.25	71.25 ± 0.81	16.74 ± 0.04 (16.78)
	1:0.2:1	0.55 ± 0.00	22.68 ± 0.30	66.78 ± 2.39	14.48 ± 0.68 (15.27)
	0:1:1	0.48 ± 0.00	22.41 ± 0.84	64.73 ± 3.52	12.08 ± 0.22 (12.29)

**Table 2:** Device metrics of PM6:BT-CIC:IT-4F ternary blends at different composition ratios under AM 1.5G and TL84 indoor illumination. The reported averages (mean ± standard deviation) were obtained from 10 devices.

Light Source	Ratio	V_OC_ (V)	J_SC_ (mA/cm^2^)	FF (%)	PCE (%) (Max)
Sun	1:0:1.2	0.86 ± 0.01	17.44 ± 0.18	57.82 ± 1.96	8.67 ± 0.28 (8.75)
	1:0.2:1	0.85 ± 0.00	17.69 ± 0.27	57.20 ± 0.81	8.56 ± 0.11 (8.68)
	1:0:1.2	0.81 ± 0.01	19.86 ± 0.29	44.75 ± 3.84	7.18 ± 0.75 (8.13)
**Light Source**	**Ratio**	**V_OC_** **(V)**	**J_SC_** **(μA/cm^2^)**	**FF** **(%)**	**PCE** **(%) (Max)**
TL84	1:0:1.2	0.65 ± 0.00	22.27 ± 0.04	61.97 ± 0.48	15.43 ± 0.03 (15.45)
	1:0.2:1	0.65 ± 0.00	19.30 ± 0.79	64.40 ± 3.01	13.88 ± 0.30 (14.28)
	1:1.2:0	0.62 ± 0.01	27.53 ± 1.75	45.72 ± 2.64	13.48 ± 1.35 (14.74)

**Table 3:** Comparative summary of mechanistic features, recombination behavior, and device-level outcomes in D–D–A and D–A–A ternary organic photovoltaic systems.

Aspect	D–D–A Systems	D–A–A Systems	General Implication
Spectral behavior	Additional donor absorption overlaps with the host donor, creating redundancy without net EQE gain.	Secondary acceptor extends NIR absorption, but exciton harvesting is inefficient, leading to suppressed EQE.	Broadened absorption seldom translates into useful photocurrent.
Energetic alignment	Similar HOMO levels between donors reduce energetic offsets and narrow the driving force for exciton dissociation.	LUMO offsets between acceptors are mismatched, generating non-ideal CT states and energetic disorder.	Both cases destabilize the energetic landscape, lowering VOC.
Exciton dynamics	Competing donor–donor transfer routes extend exciton lifetimes and enhance geminate/trap-assisted recombination.	Competing acceptor–acceptor electron-transfer channels create recombination-prone CT states.	Multiple parallel pathways amplify recombination losses.
Charge transport	Donor–donor mixing disrupts percolation networks, producing mobility asymmetry between electrons and holes.	Secondary acceptor interrupts electron percolation, prolonging extraction time and lowering mobilities.	Transport imbalance consistently reduces FF and JSC.
Morphology	AFM shows smooth surfaces, no large-scale segregation; degradation arises from subtle miscibility issues between donors.	AFM shows smooth surfaces, no large-scale segregation; decline originates from molecular-level miscibility limits between acceptors.	Morphology is not the dominant factor; molecular-scale effects are critical.
Light-intensity dependence	n > 1 and α < 1 indicate combined trap-assisted and bimolecular recombination, magnified under TL84.	Same recombination signatures, often more severe under TL84 due to low photon flux and longer carrier lifetimes.	Indoor lighting amplifies recombination penalties in both strategies.
Device performance	Declines in VOC, JSC, and FF under both AM 1.5G and TL84; binary endpoints outperform ternary blends.	Similar declines; ternary PCE consistently lower than binary references across all conditions.	Adding a third component does not guarantee performance gains.

## Data Availability

The original contributions presented in this study are included in the article/Supplementary Materials. Further inquiries can be directed to the corresponding author.

## Supplementary Materials

The following supporting information can be downloaded at: https://www.mdpi.com/article/10.3390/nano15221702/s1, Table S1: Light-intensity dependence of devices with different PM6:PBDB-T:ITIC-4F ratios under various illumination conditions; Table S2: Charge transport analysis of PM6:PBDB-T:IT-4F devices with different ratios under various light sources; Table S3: Photovoltaic parameters of devices with different PCDTBT:PM6:PC_71_BM ratios under various illumination conditions; Table S4: Light-intensity dependence of devices with different PM6:BT-CIC:IT-4F ratios under various illumination conditions; Table S5: Charge-transport analysis of devices with different PM6:BT-CIC:IT-4F ratios under various illumination conditions; Table S6: Photovoltaic parameters of devices with different PM6:Y6:PC_71_BM ratios under various illumination conditions; Table S7: Photovoltaic parameters of devices with different PTB7-Th:3TT-FIC:PC_71_BM ratios under various illumination conditions; Figure S1: (a) Energy level diagram and (b) absorption spectra of the active layer materials PCDTBT, PM6, and PC_71_BM. J–V characteristics of devices with various PCDTBT:PM6:PC_71_BM ratios under (c) solar illumination and (d) indoor light; Figure S2: J–V characteristics of devices with different PM6:Y6:PC_71_BM ratios under (a) solar illumination and (b) indoor light; Figure S3: (a) Energy level diagram of donor/acceptor materials, (b) absorption spectra of donor and acceptor materials, and (c) absorption spectra of blends with different donor/acceptor ratios; Figure S4: J–V characteristics of devices with various PTB7-Th:3TT-FIC:PC_71_BM ratios under (a) standard solar illumination and (b) TL84 indoor light (200 lux).
