# Supplementary material for: Ternary Organic Photovoltaics at a Turning Point: Mechanistic Perspectives on Their Constraints
Source: Nanomaterials (Basel). 2025 Nov 11;15(22):1702. doi: 10.3390/nano15221702 (PMC12655043; doi:10.3390/nano15221702)
Supplement: Supplementary file 1 [file nanomaterials-15-01702-s001.zip › nanomaterials-3951488-supplementary.pdf]

Table S1. Light-intensity dependence of devices with different PM6:PBDB-T:ITIC-4F ratios under various illumination conditions.

Light source	Ratio	n	α
**Sun**	1:0:1	1.548	0.937
	1:0.2:1	1.697	0.947
	0:1:1	1.605	0.928
**TL84**	1:0:1	1.307	0.966
	1:0.2:1	1.453	0.964
	0:1:1	1.370	0.964

Table S2. Charge transport analysis of PM6:PBDB-T:IT-4F devices with different ratios under various light sources.

Light source	Ratio	Charge extraction time (μs)	Charge decay time (μs)	Mobility ($\times10^{-5}$ cm^2/Vs)
**Sun**	1:0:1	0.66 ±0.01	13.83 ±0.00	3.50 ±0.36
	1:0.2:1	1.90 ±0.00	18.20 ±2.71	1.25 ±0.43
	0:1:1	1.16 ±0.30	12.07 ±5.40	1.91 ±0.93
**TL84**	1:0:1	2.78 ±0.06	17.20 ±8.88	21.45 ±7.00
	1:0.2:1	4.36 ±0.10	24.21 ±.3.61	2.90 ±1.12
	0:1:1	3.47 ±0.23	15.88 ±9.48	7.41 ±3.61

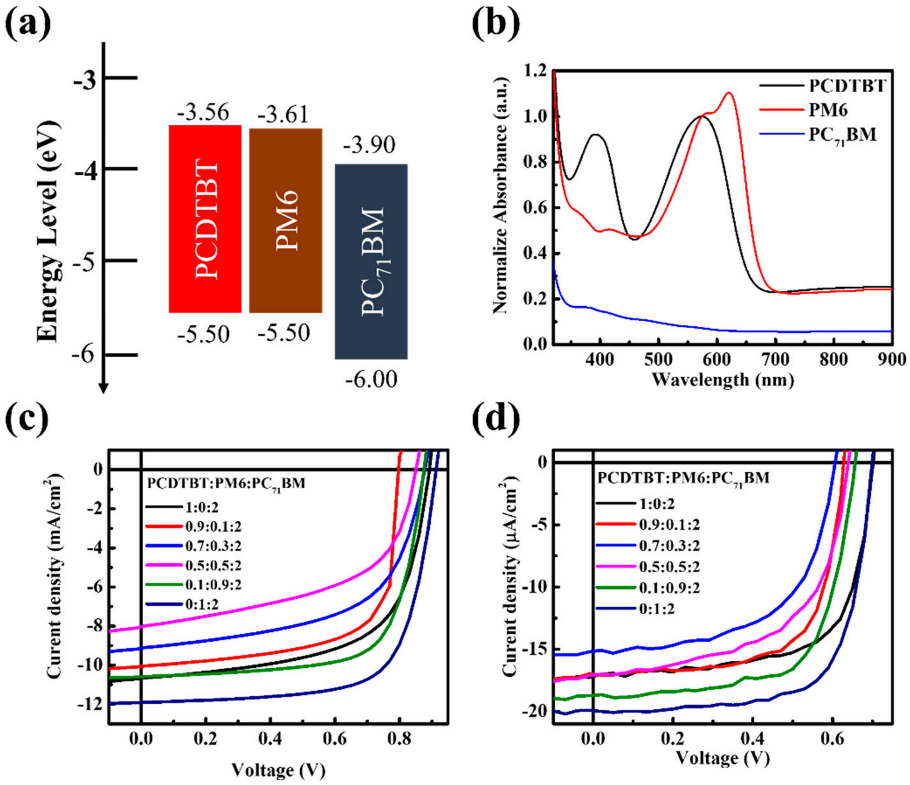

Figure S1. (a) Energy level diagram and (b) absorption spectra of the active layer materials PCDTBT, PM6, and $PC_{71}BM$. J–V characteristics of devices with various PCDTBT:PM6:$PC_{71}BM$ ratios under (c) solar illumination and (d) indoor light.

Table S3. Photovoltaic parameters of devices with different PCDTBT:PM6:$PC_{71}BM$ ratios under various illumination conditions.

Light source	Ratio	V_{OC} (V)	J_{SC} (mA/cm^2)	FF (%)	PCE(%) (Max)
**SUN**	1:0:2	0.90 ±0.00	10.41 ±0.15	61.84 ±0.23	5.77 ±0.09 **(5.91)**
	0.9:0.1:2	0.87 ±0.00	10.31 ±0.19	62.78 ±1.05	5.65 ±0.12 **(5.78)**
	0.7:0.3:2	0.88 ±0.01	9.01 ±0.27	55.96 ±1.59	4.43 ±0.23 **(4.73)**
	0.5:0.5:2	0.85 ±0.00	7.80 ±0.25	53.15 ±0.50	3.51 ±0.10 **(3.62)**
	0.1:0.9:2	0.87 ±0.01	10.53 ±0.10	68.48 ±2.45	6.24 ±0.27 **(6.48)**
	0:1:2	0.90 ±0.00	11.52 ±0.10	70.41 ±1.65	7.32 ±0.15 **(7.49)**
	**Ratio**	**V_{OC} (V)**	**J_{SC} (μA/cm^2)**	**FF (%)**	**PCE(%) (Max)**
**TL 84 (200 lux)**	1:0:2	0.67 ±0.01	19.92 ±0.32	62.44 ±2.51	14.28 ±0.75 **(14.91)**
	0.9:0.1:2	0.61 ±0.02	19.76 ±0.81	58.82 ±1.60	12.23±0.96 **(13.55)**
	0.7:0.3:2	0.57 ±0.01	16.87 ±0.53	54.67±0.65	9.13 ±0.23 **(9.17)**
	0.5:0.5:2	0.57 ±0.00	14.63 ±1.03	52.53 ±0.94	7.58 ±0.66 **(8.05)**
	0.1:0.9:2	0.65 ±0.00	18.15 ±0.49	67.58 ±1.71	13.78 ±0.61 **(14.45)**
	0:1:2	0.70 ±0.00	19.35 ±0.89	69.89 ±1.27	16.27 ±0.86 **(16.97)**

Table S4. Light-intensity dependence of devices with different PM6:BT-CIC:IT-4F ratios under various illumination conditions.

Light source	Ratio	n	α
Sun	1:0:1.2	1.951	0.901
	1:0.2:1	1.900	0.953
	1:1.2:0	1.677	0.950
TL84	1:0:1.2	1.122	0.960
	1:0.2:1	1.236	0.966
	1:1.2:0	1.139	0.992

Table S5. Charge-transport analysis of devices with different PM6:BT-CIC:IT-4F ratios under various illumination conditions.

Light source	Ratio	Charge extraction time (μs)	Charge decay time (μs)	Mobility ($\times 10^{-5}$ cm^2/Vs)
Sun	1:0:1	1.27 ±0.01	26.62 ±0.00	0.78 ±0.13
	1:0.2:1	1.52 ±0.22	26.62 ±3.97	0.40 ±0.05
	0:1:1	1.50 ±0.00	28.13 ±2.93	1.59 ±0.09
TL84	1:0:1	3.01 ±0.38	26.78 ±1.40	5.62± 0.37
	1:0.2:1	5.66 ±0.57	26.48 ±1.98	2.29 ±0.40
	0:1:1	3.26 ±1.00	21.48 ±3.45	8.16 ±1.70

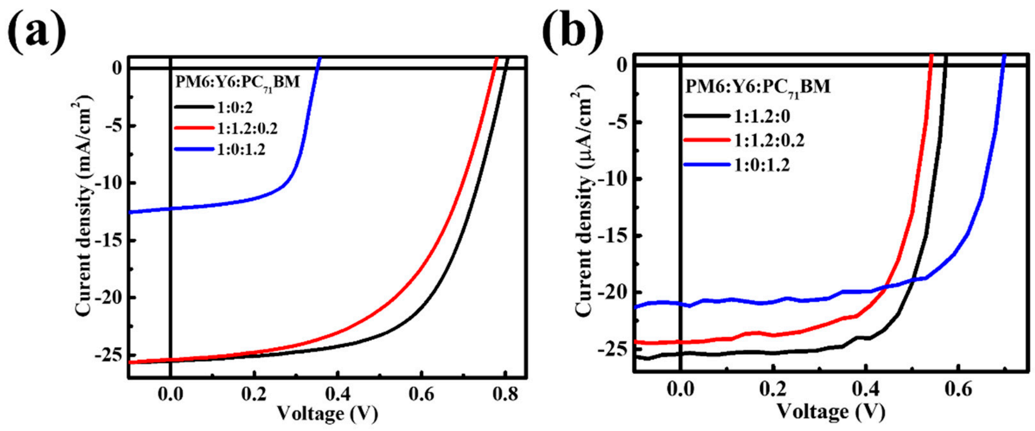

Figure S2. J–V characteristics of devices with different PM6:Y6:$PC_{71}BM$ ratios under (a) solar illumination and (b) indoor light.

Table S6. Photovoltaic parameters of devices with different PM6:Y6:$PC_{71}BM$ ratios under various illumination conditions.

**Light source**	**Ratio**	**V_{OC} (V)**	**J_{SC} (mA/cm^2)**	**FF (%)**	**PCE(%) (Max)**
**SUN**	1:1.2:0	0.79 ±0.01	24.89 ±0.47	58.58 ±1.94	11.53 ±0.69 **(12.52)**
	1:1.2:0.2	0.78 ±0.00	25.40 ±0.33	52.58 ±1.65	10.37 ±0.34 **(10.73)**
	1:0:1.2	0.90 ±0.02	12.03 ±0.25	58.93 ±6.43	6.40 ±0.68 **(7.00)**
	**Ratio**	**V_{OC} (V)**	**J_{SC} ($\mu A/cm^2$)**	**FF (%)**	**PCE (%) (Max)**
**TL 84 (200 lux)**	1:1.2:0	0.57 ±0.00	25.33 ±0.07	70.62 ±0.72	17.64 ±0.22 **(17.79)**
	1:1.2:0.2	0.54 ±0.00	24.03 ±0.84	65.14 ±1.48	14.50 ±0.62 **(15.04)**
	1:0:1.2	0.69 ±0.01	21.11 ±0.38	66.14 ±0.71	16.68 ±0.47 **(17.22)**

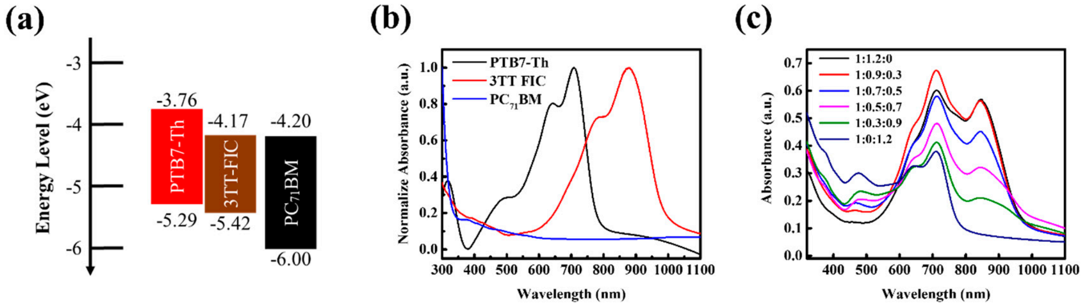

Figure S3. (a) Energy level diagram of donor/acceptor materials, (b) absorption spectra of donor and acceptor materials, and (c) absorption spectra of blends with different donor/acceptor ratios.

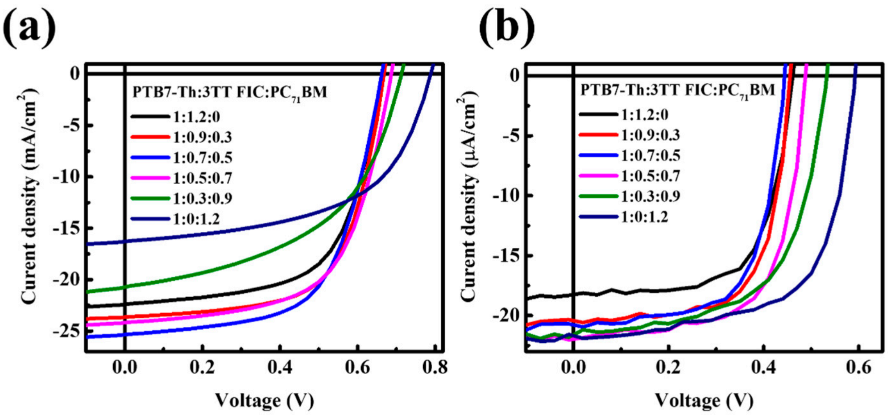

Figure S4. J–V characteristics of devices with various PTB7-Th:3TT-FIC:$PC_{71}BM$ ratios under (a) standard solar illumination and (b) TL84 indoor light (200 lux).

Table S7. Photovoltaic parameters of devices with different PTB7-Th:3TT-FIC:$PC_{71}BM$ ratios under various illumination conditions.

**Light source**	**Ratio**	**V_{OC} (V)**	**J_{SC} (mA/cm^2)**	**FF (%)**	**PCE (%) (Max)**
**SUN**	1:1.2:0	0.66 ±0.01	22.28 ±0.23	61.11 ±1.40	8.97 ±0.34 **(9.24)**
	1:0.9:0.3	0.66 ±0.00	23.95 ±0.25	62.12 ±1.99	9.88 ±0.34 **(10.13)**
	1:0.7:0.5	0.66 ±0.00	25.28 ±0.14	60.15 ±1.21	10.02 ±0.21 **(10.32)**
	1:0.5:0.7	0.69±0.00	23.97 ±0.42	57.05 ±4.17	9.45 ±0.78 **(10.12)**
	1:0.3:0.9	0.71 ±0.00	20.53 ±0.20	49.99 ±0.35	7.29 ±0.09 **(7.37)**
	1:0:1.2	0.78 ±0.01	15.98 ±0.21	55.57 ±0.27	6.88 ±0.17 **(7.11)**
**TL 84 (200 lux)**	**Ratio**	**V_{OC} (V)**	**J_{SC} (μA/cm^2)**	**FF (%)**	**PCE (%) (Max)**
	1:1.2:0	0.46 ±0.01	17.59 ±0.35	64.51 ±2.55	9.05 ±0.52 **(9.74)**
	1:0.9:0.3	0.45 ±0.00	19.30 ±1.35	67.00 ±2.17	10.13 ±0.71 **(10.90)**
	1:0.7:0.5	0.44 ±0.00	20.57 ±0.38	64.06 ±1.58	10.08 ±0.33 **(10.58)**
	1:0.5:0.7	0.49 ±0.00	20.83 ±1.28	65.22 ±1.11	11.43 ±0.83 **(12.19)**
	1:0.3:0.9	0.53 ±0.00	20.56 ±0.79	60.99 ±1.35	11.50 ±0.61 **(12.01)**
	1:0:1.2	0.59 ±0.00	21.95 ±0.36	61.71 ±2.38	13.84 ±0.64 **(14.41)**
